# Supplementary material for: Distinct clinical phenotypes and their neuroanatomic correlates in chronic traumatic brain injury
Source: Brain Commun. 2025 Jun 6;7(3):fcaf216. doi: 10.1093/braincomms/fcaf216 (PMC12198765; doi:10.1093/braincomms/fcaf216)
Supplement: fcaf216_Supplementary_Data [file fcaf216_supplementary_data.docx]

# **Supplementary Table 1:** Details on lesion correction ratings.

| RATING | ASEG | SURFACE |
| --- | --- | --- |
| 1 | Cortical GM/WM segmentation is free of minor errors, subcortical and cerebellar contrast is not oversaturated, subcortical and cerebellar segmentations are free of minor errors and deviations | Pial and white surfaces closely follow anatomical boundaries, no minor areas excluded from either |
| 2 | Minor deviations observable in GM/WM segmentations, contrast in cerebellar and subcortical areas may be difficult to assess, cerebellar and subcortical segmentations appear reasonable with some over and under-labeling. Requires minor manual edits | Pial and white surfaces may exclude some parts of the cortex, but no major exclusions are observable. Requires minor manual edits |
| 3 | Obvious deviations observable in GM/WM segmentations, slightly worse than rating '2' and requiring major manual edits | Obvious exclusions on the surfaces requiring major manual edits, but not enough to warrant exlusion |
| 4 | EXCLUDE - Major deviations observable in GM/WM segmentations, little to no contrast in subcortical and cerebellar areas, major deviations in subcortical and cerebellar labels | EXCLUDE - Major disruptions in pial and white surfaces, excludes and/or mislabels areas of cortex |

**Supplemental Table 2:** Characteristics of the sample by training and validation set

|  | Training (n=195) | Validation (n=86) |
| --- | --- | --- |
| Age, Mean (SD) | 58.1 (16.8) | 56.3 (13.9) |
| Age group, n (col %)  <40  40-54  55-64  65+ | 31 (15.9%)  47 (24.1%)  46 (23.6%)  71 (36.4%) | 9 (10.5%)  31 (36.1%)  21 (24.4%)  25 (29.1%) |
| Sex, Female (col %) | 74 (38.0%) | 22 (26.0%) |
| Education, n (col %)  <College  ≥College degree | 52 (26.7%)  143 (73.3%) | 25 (29.1%)  61 (70.9%) |
| Race, n (%)  White  Black  Other | 168 (86.2%)  15 (7.7%)  12 (6.2%) | 74 (86.1%)  4 (4.7%)  8 (9.3%) |
| Hispanic ethnicity, n (col %) | 13 (6.7%) | 10 (11.6%) |
| Marital status, n (col %)  Never married  Married/partnered  Divorced/widowed | 56 (28.7%)  78 (40.0%)  61 (31.3%) | 17 (19.8%)  43 (50.0%)  26 (30.2%) |
| Employment, n (col %)  Working/Student  Unemployed  Retired  Disabled  Other | 65 (33.3%)  12 (6.2%)  60 (30.8%)  45 (23.1%)  13 (6.7%) | 28 (32.6%)  6 (7.0%)  25 (29.1%)  23 (26.7%)  4 (4.7%) |
| Years since most recent TBI, median (IQR) | 7.4 (3.3-17.3) | 9.9 (4.1-18.8) |
| Years since first TBI, median (IQR) | 28.0 (12.9-48.3) | 26.9 (11.0-44.0) |
| Injury severity of most severe lifetime injury, n (%)  Blow to head only without  any LOC/DAC  Mild TBI  Moderate TBI  Severe TBI | 1 (0.5%)  58 (29.9%) 30 (15.5%)  105 (54.1%) | 1 (1.2%)  18 (21.2%)  4 (4.7%)  62 (72.9%) |

# **Supplementary Table 3:** Rank Order of Top 12 Contributors and Loadings of Individual Measures Making up Principal Components (PC1-PC3)

| Rank order | PC1 Measures (24.8% variance) | PC1 loadings | PC2 Measures (14.5% variance) | PC2 loadings | PC3 Measures (6.0% variance) | PC3 loadings |
| --- | --- | --- | --- | --- | --- | --- |
| 1 | RAND-Social Function | 0.727 | CVLT-SDFR | 0.692 | UPDRS-Part 3 Total | 0.609 |
| 2 | RAND-Emotional Wellbeing | 0.693 | CVLT-Immediate Recall | 0.688 | RAND Physical Function | -0.486 |
| 3 | RAND-Energy/Fatigue | 0.670 | CVLT-LDFR | 0.677 | Sit-to-stand | 0.456 |
| 4 | QOL-Social | 0.669 | WMS-LM2 | 0.590 | RAND-Emotional Limitations | 0.405 |
| 5 | SWLS | 0.668 | WMS-LM1 | 0.551 | CVLT-Semantic Clustering | 0.358 |
| 6 | QOL-Anxiety | -0.667 | QOL-Fatigue | 0.499 | BIS-Motor | -0.299 |
| 7 | QOL-Fatigue | -0.644 | RAND-Energy/Fatigue | -0.493 | Trails A | 0.289 |
| 8 | QOL-Depression | -0.641 | REY-Delay Recall | 0.469 | Assist-Illicit Substance | -0.283 |
| 9 | WAIS Coding | 0.635 | REY-Immediate Recall | 0.450 | Grip strength-Dominant Hand | -0.283 |
| 10 | MIDUS Health | -0.628 | Symbol search | 0.440 | CVLT-SDFR | 0.274 |
| 11 | RAND-General Health | 0.606 | RAND-General Health | -0.433 | Assist-Alcohol | -0.270 |
| 12 | Symbol search | 0.599 | COWAT-Animals | 0.430 | RAND-Pain | -0.262 |

# **Supplementary Table 4:** Cluster Validity Indices

|  | Connectivity | Dunn Index | Silhouette |
| --- | --- | --- | --- |
| 3 group | 25.765 | 0.095 | 0.328 |
| 4 group | 39.06 | 0.099 | 0.320 |
| Optimal number of clusters based on given index | 3 group | 4 group | 3 group |

# **Supplementary Table 5:** Characteristics of the sample with and without MRI

|  | Without MRI (n=99) | With MRI (n=182) | p-value |
| --- | --- | --- | --- |
| Age, Mean (SD) | 56.2 (17.6) | 58.3 (15.0) | 0.296 |
| Age group, n (col %)  <40  40-54  55-64  65+ | 19 (19.2%)  27 (27.3%)  20 (20.2%)  33 (33.3%) | 21 (11.5%)  51 (28.0%)  47 (25.8%)  63 (34.6%) | 0.317 |
| Sex, Female (col %) | 38 (38.4%) | 58 (31.9%) | 0.271 |
| Education, n (col %)  <College  ≥College degree | 23 (23.2%)  76 (76.8%) | 54 (29.7%)  128 (70.3%) | 0.248 |
| Race, n (%)  White  Black  Other | 82 (82.8%) 8 (8.1%) 9 (9.1%) | 160 (87.9%) 11 (6.0%)  11 (6.0%) | 0.491 |
| Hispanic ethnicity, n (col %) | 7 (7.1%) | 16 (8.8%) | 0.615 |
| Marital status, n (col %)  Never married  Married/partnered  Divorced/widowed | 29 (29.3%)  45 (45.5%)  25 (25.3%) | 44 (24.2%)  76 (41.8%) 62 (34.1%) | 0.293 |
| Employment, n (col %)  Working/Student  Unemployed  Retired  Disabled  Other | 33 (33.3%)  8 (8.1%)  27 (27.3%)  28 (28.3%)  3 (3.0%) | 60 (33.0%)  10 (5.5%)  58 (31.9%)  40 (22.0%) 14 (7.7%) | 0.345 |

**Supplementary Table 6:** Pairwise comparisons of least squares mean difference in cortical network volume by cluster^¥^

| **Pairwise comparison** | **Network** | **Least Squares** **Mean Difference (95% CI)** | **p-value** |
| --- | --- | --- | --- |
| **Cluster 1_mixed_ – Cluster 2_cognitive_** | Executive control | 0.59 (-0.08, 1.25) | 0.103 |
|  | Dorsal attention | 0.35 (-0.30, 1.01) | 0.495 |
|  | Limbic | 0.89 (0.17, 1.60) | 0.008* |
|  | Default mode network | 0.64 (-0.04, 1.31) | 0.073 |
|  | Somatomotor | 0.34 (-0.31, 1.00) | 0.525 |
|  | Salience | 0.23 (-0.44, 0.90) | 0.808 |
|  | Visual | 0.46 (-0.18, 1.10) | 0.245 |
| **Cluster 1_mixed_ – Cluster 3_mood/behavior_** | **Network** | **Least Squares** **Mean Difference (95% CI)** | **p-value** |
|  | Executive control | 0.34 (-0.28, 0.96) | 0.483 |
|  | Dorsal attention | 0.30 (-0.31, 0.91) | 0.575 |
|  | Limbic | 0.75 (0.08, 1.41) | 0.021* |
|  | Default mode network | 0.37 (-0.26, 1.00) | 0.417 |
|  | Somatomotor | 0.20 (-0.41, 0.82) | 0.821 |
|  | Salience | 0.21 (-0.41, 0.84) | 0.812 |
|  | Visual | 0.34 (-0.26, 0.93) | 0.452 |
| **Cluster 2_cognitive_ – Cluster 3_mood/behavior_** | **Network** | **Least Squares** **Mean Difference (95% CI)** | **p-value** |
|  | Executive control | -0.25 (-0.76, 0.25) | 0.593 |
|  | Dorsal attention | -0.05 (-0.56, 0.45) | 0.993 |
|  | Limbic | -0.14 (-0.69, 0.41) | 0.914 |
|  | Default mode network | -0.26 (-0.79, 0.26) | 0.555 |
|  | Somatomotor | -0.14 (-0.65, 0.37) | 0.891 |
|  | Salience | -0.02 (-0.54, 0.50) | 0.959 |
|  | Visual | -0.12 (-0.61, 0.37) | 0.919 |

^¥^: Pairwise comparisons with Cluster 4 (fewest deficits) not included in this table because it is included in Table 3; A visual of these least squares mean differences by cluster group is provided in Figure 5.

# **Supplementary Fig 1:** Exemplar of MRI scan before and after implementation of novel lesion correction methodology

**
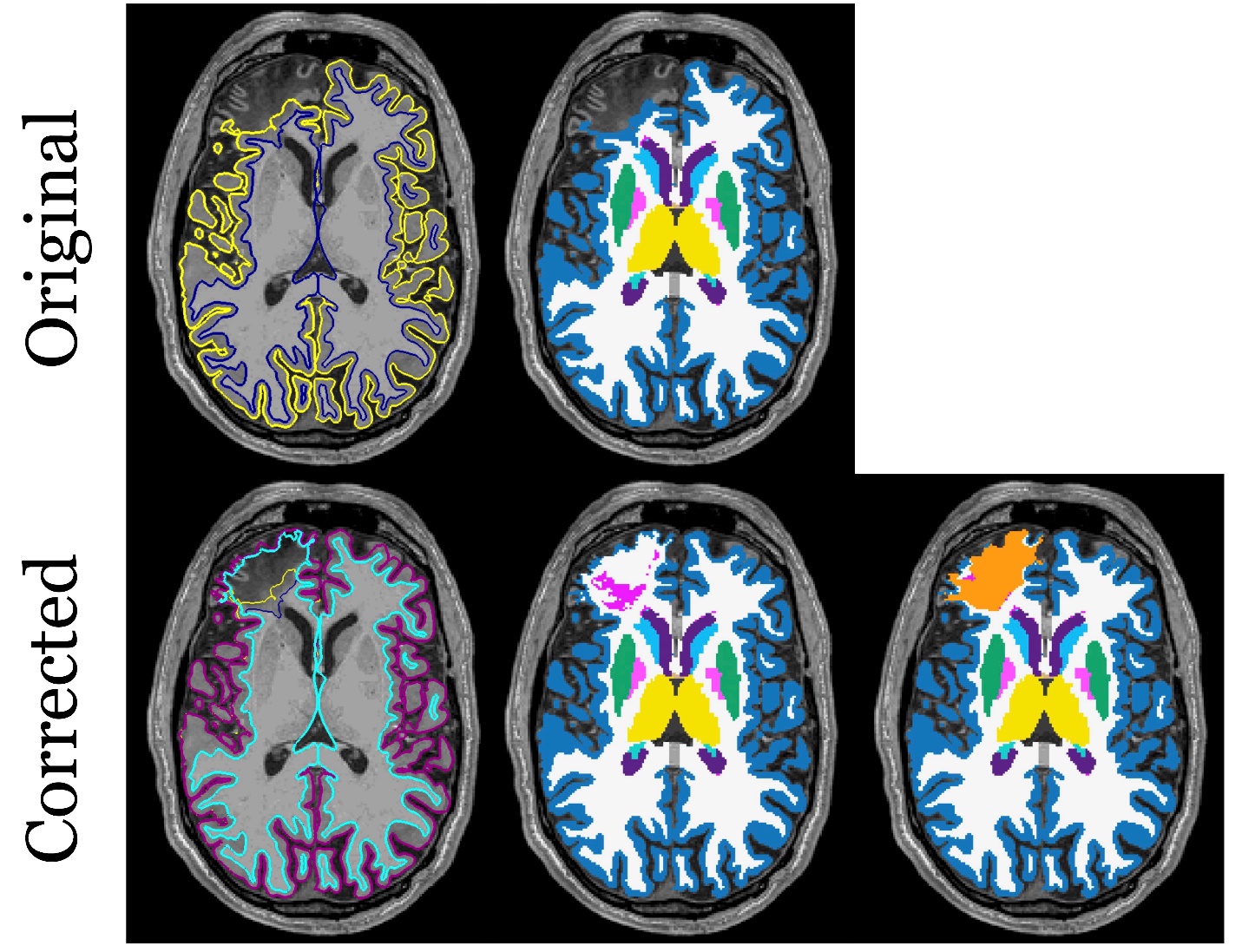
**

Supplementary Fig 1 caption: The “before” and “after” Magnetic Resonance Imaging (MRI) scans illustrating the implementation of the novel lesion correction methodology.

# **Supplementary Fig 2:** Initial cutpoint selected by the Hierarchical Clustering on Principal Components (HCPC) algorithm


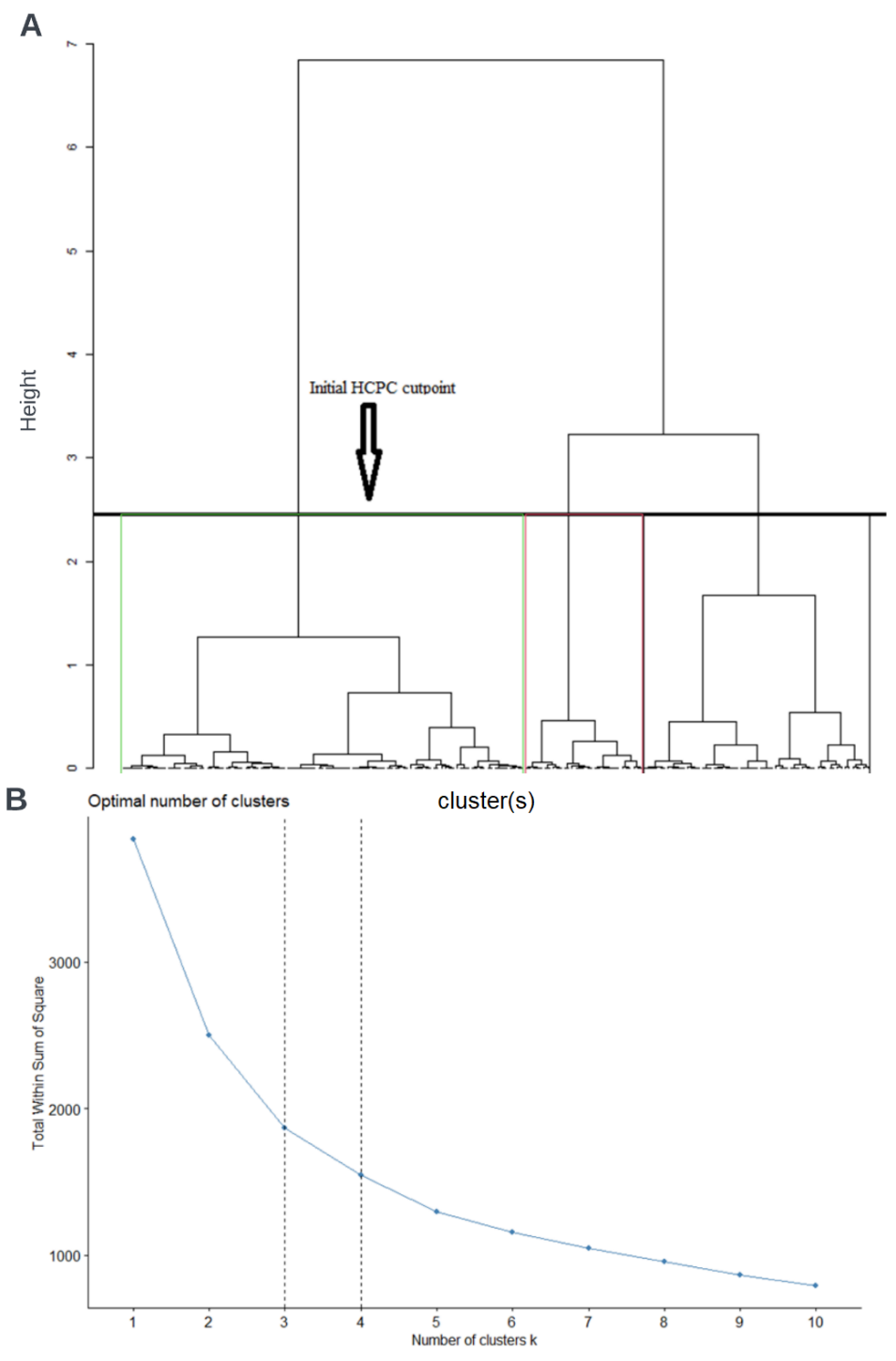


Supplementary Fig 2 caption: The two panels here were based on the Hierarchical Clustering on Principal Components (HCPC) model that was applied to the training dataset (n=195). 1A) The algorithm outputted an initial cutpoint shown here. The algorithm suggested 3 components, which we tested empirically to assess reliability and validity versus other specifications. 1B) The total within cluster sum of squares is a measure of variance within cluster. Each dot represents the total within sum of square for each increasing number of cluster. There was an elbow at 3 and 4 cluster specifications, which were further empirically compared. There were no between group statistics and associated p-values for this figure.

**Supplemental Fig 3**: Hierarchical cluster group assignment (validation set)


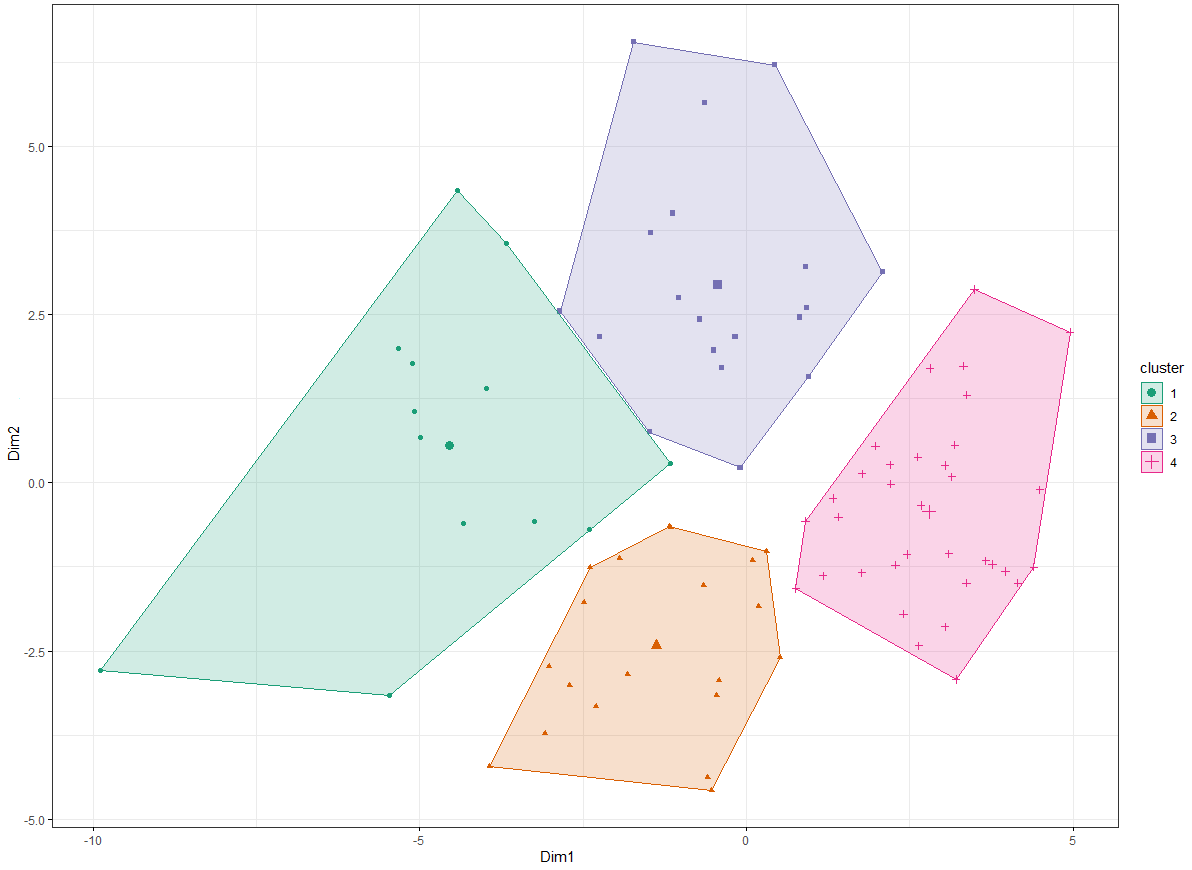


Supplemental Fig 3 caption: Hierarchical cluster group assignment in the validation set (n=86). The results are based on the 4-cluster Hierarchical Clustering on Principal Components (HCPC) model. Here, each dot represents a participant in the validation sample. Participants are depicted in the x-y coordinate space based on their PC1 vs. PC2 scores. The cluster membership of each participant is color-coded. There were no between group statistics and associated p-values for this figure.

# **Supplementary Fig 4:** Three group cluster classification in training (Panel A) and validation (Panel B) sets


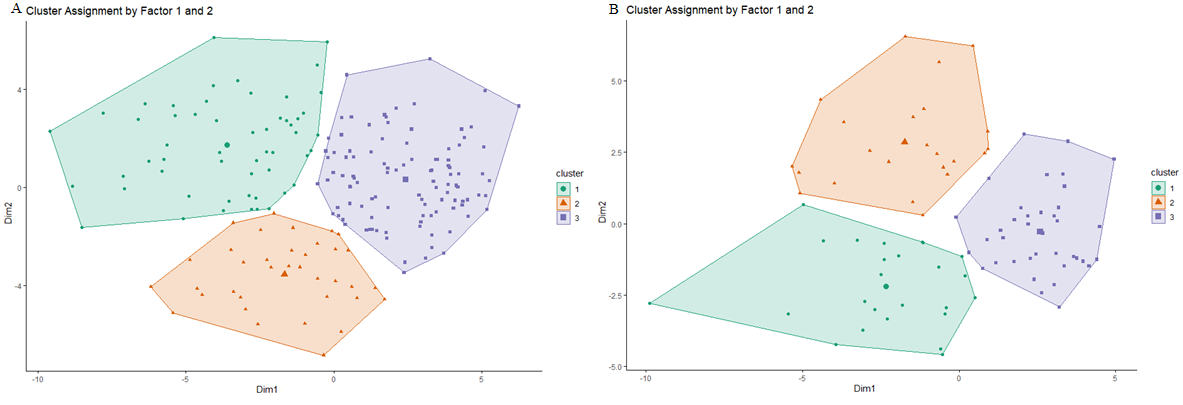


Supplementary Fig 4 caption: The three-cluster Hierarchical Clustering on Principal Components (HCPC) model run in the training (n=195) (panel A) and validation (n=86) (panel B) sets. There was poor reliability of cluster centroids in the internal validation versus the training sets; therefore, the three-cluster specification was deemed to have unacceptable reliability. There were no between group statistics and associated p-values for this figure.

**Supplemental Fig 5**: Heat map characterizing average values of neurobehavioral measures by cluster assignment (validation set; n=86)


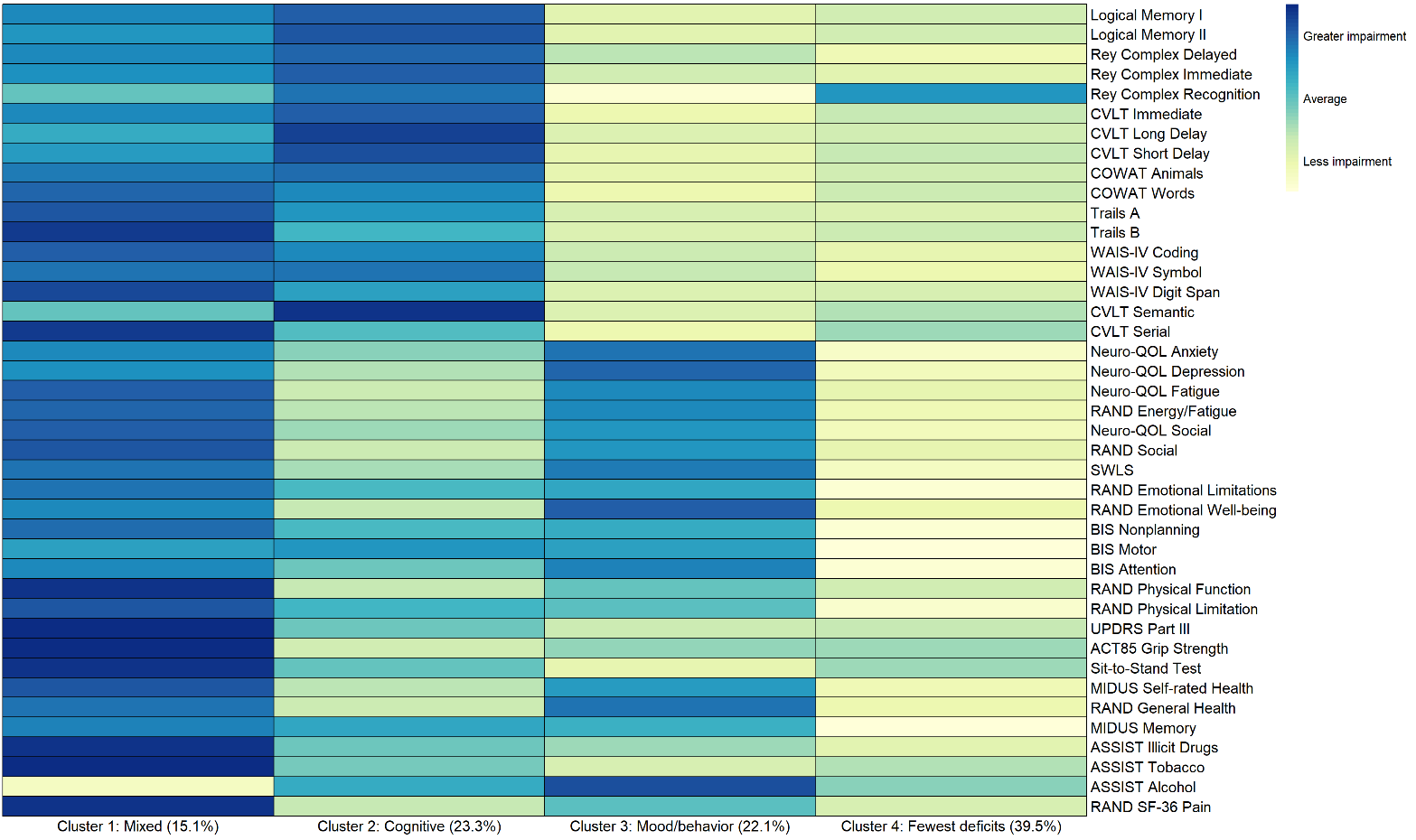


Supplemental Fig 5 caption: Heat map characterizing average values of neurobehavioral measures by cluster assignment in the validation set. The measures were transformed such that darker colors represent greater impairment, and lighter colors represent less impairment. The qualitative descriptors are consistent as we observed in the training set: Cluster 1: Mixed trait deficits; Cluster 2) Predominant cognitive deficits; Cluster 3: Predominant mood and behavioral deficits; Cluster 4: Relatively few deficits. No between-group statistical tests and p-values were derived for the heat maps.

**Supplementary Fig 6:** Sensitivity analysis: replication of cluster analysis among only participants who have an MRI

**
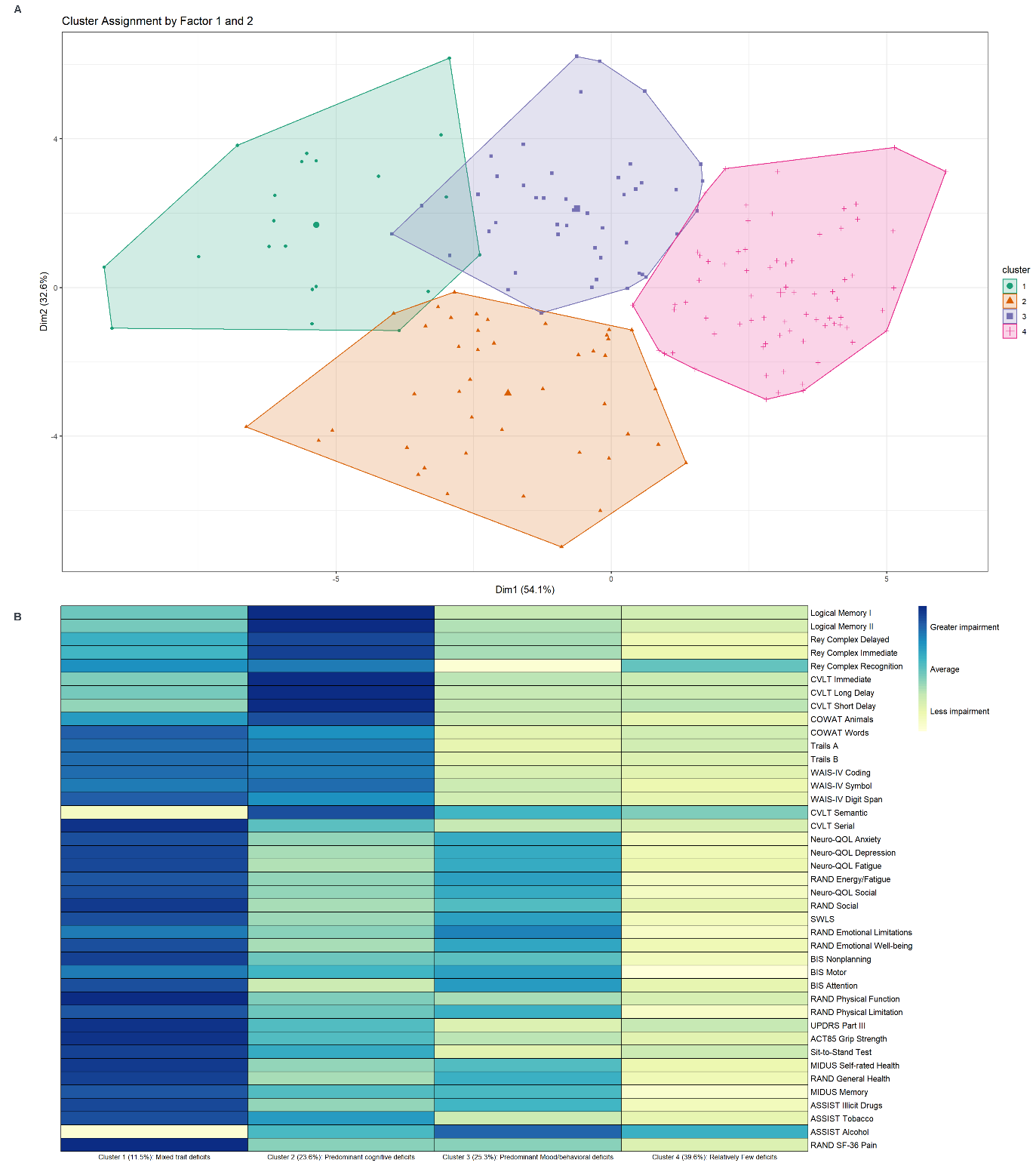
**

Supplementary Fig 6 caption: Panel A) is the four-group cluster grouping as specified in the primary analysis in the whole sample. Analyses were run only among n=182 participants with MRI to assess the reliability of primary results among this subgroup to determine if results are generalizable. The sample size for each cluster in the MRI sample was as follows: Cluster 1 (mixed trait) n=19, Cluster 2 (Predominant cognitive) n=37, Cluster 3 (Predominant mood/behavioral) n=52, Cluster 4 (Relatively few deficits) n=74. Each data point in Panel A represents individual participants in the sample, color coded by cluster assignment. Panel B) is the heat map based on the cluster groupings in Panel A among n=182 participants with MRI. The sample size for each cluster is the same as described above in panel A. Each cell represents the mean value for a given measure for a given cluster group, and darker values represent more impairment relative to other cluster groups. We determined the results are largely generalizable to the findings from Aim 1. No between-group statistical tests and p-values were derived for the heat maps.

# **Supplementary Fig 7:** Raw (unadjusted) mean volumes by cluster and network


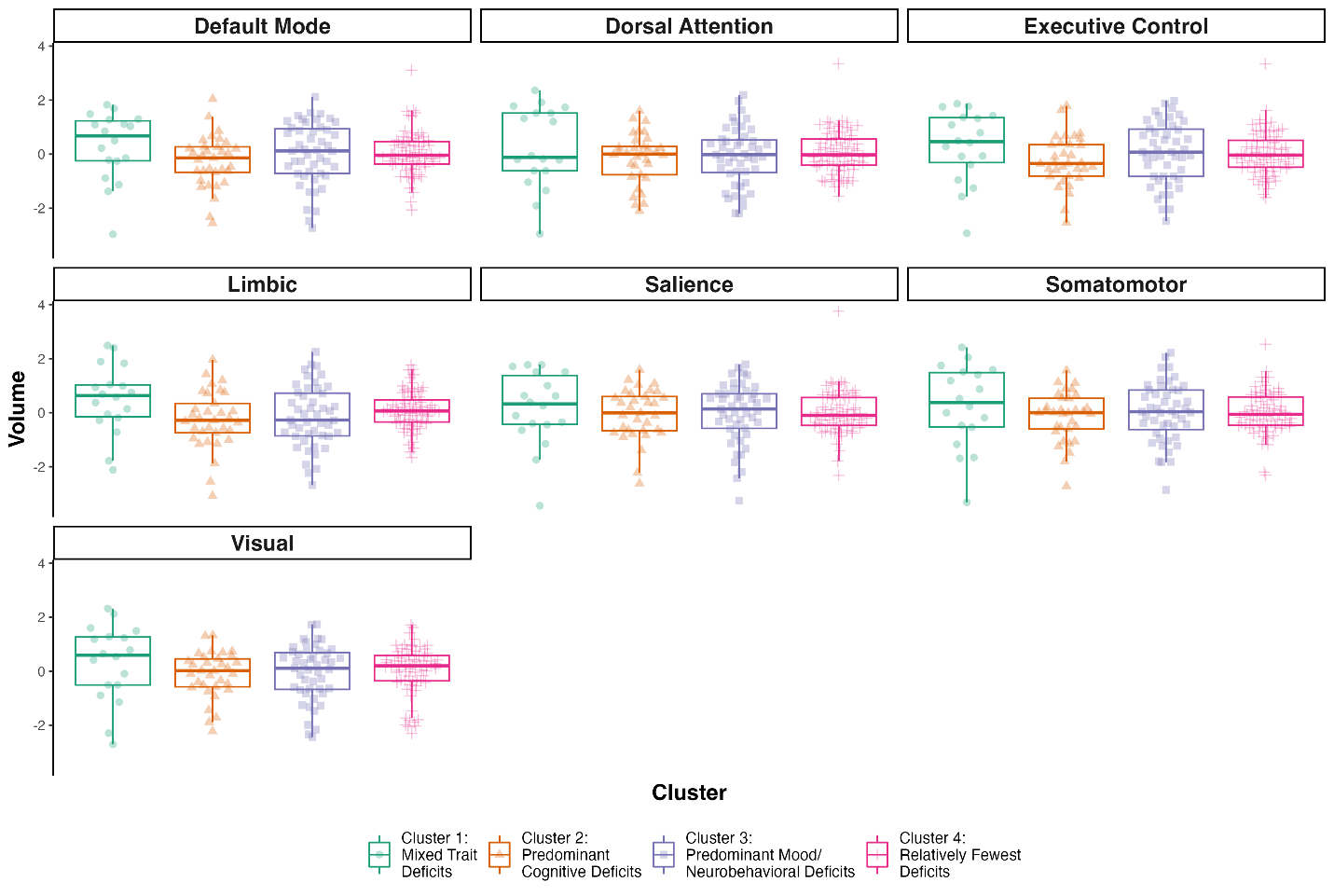


Supplementary Fig 7 caption: Unadjusted (raw) mean network volumes for each of the 7 networks from the Yeo-7 atlas by phenotype group. The graph was conducted among n=182 participants with MRI data. The sample size for each cluster in the MRI sample was as follows: Cluster 1 (mixed trait) n=19, Cluster 2 (Predominant cognitive) n=37, Cluster 3 (Predominant mood/behavioral) n=52, Cluster 4 (Relatively few deficits) n=74. Each dot represents individual participants, and the bar graphs are cluster group level statistics. These unadjusted data are descriptive; no between-group statistical tests were performed for this figure (only the adjusted models).
